# Autophagy-induced RelB/p52 activation mediates tumour-associated macrophage repolarisation and suppression of hepatocellular carcinoma by natural compound baicalin

**DOI:** 10.1038/cddis.2015.271

**Published:** 2015-10-22

**Authors:** H-Y Tan, N Wang, K Man, S-W Tsao, C-M Che, Y Feng

**Affiliations:** 1 School of Chinese Medicine, The University of Hong Kong, Hong Kong, China; 2 Department of Surgery, The University of Hong Kong, Hong Kong, China; 3 Department of Anatomy, The University of Hong Kong, Hong Kong, China; 4 State Key Laboratory of Synthetic Chemistry, Chemical Biology Center and Department of Chemistry, The University of Hong Kong, Hong Kong, China

## Abstract

The plasticity of tumour-associated macrophages (TAMs) has implicated an influential role in hepatocellular carcinoma (HCC). Repolarisation of TAM towards M1 phenotype characterises an immune-competent microenvironment that favours tumour regression. To investigate the role and mechanism of TAM repolarisation in suppression of HCC by a natural compound baicalin, Orthotopic HCC implantation model was used to investigate the effect of baicalin on HCC; liposome-clodronate was introduced to suppress macrophage populations in mice; bone marrow-derived monocytes (BMDMs) were induced to unpolarised, M1-like, M2-like macrophages and TAM using different conditioned medium. We observed that oral administration of baicalin (50 mg/kg) completely blocked orthotopic growth of implanted HCC. Suppression of HCC by baicalin was diminished when mice macrophage was removed by clodronate treatment. Baicalin induced repolarisation of TAM to M1-like phenotype without specific toxicity to either phenotype of macrophages. Baicalin initiated TAM reprogramming to M1-like macrophage, and promoted pro-inflammatory cytokines production. Co-culturing of HCC cells with baicalin-treated TAMs resulted in reduced proliferation and motility in HCC. Baicalin had minimal effect on derivation of macrophage polarisation factors by HCC cells, while directly induced repolarisation of TAM and M2-like macrophage. This effect was associated with elevated autophagy, and transcriptional activation of RelB/p52 pathway. Suppression of autophagy or RelB abolished skewing of baicalin-treated TAM. Autophagic degradation of TRAF2 in baicalin-treated TAM might be responsible for RelB/p52 activation. Our findings unveil the essential role of TAM repolarisation in suppressive effect of baicalin on HCC, which requires autophagy-associated activation of RelB/p52.

Liver cancer is the second most fatal cancer worldwide, with mortality rate of over 84% within 5 years.^1^ Of 782 000 cases reported worldwide in 2012, China alone has accounted for more than 50% of the total incidences. Hepatocellular carcinoma (HCC) is the major occurring liver cancers, which represents 70–85% of cases among all primary liver cancers.^2^ Our recent study postulated that poor prognosis of HCC is associated with high level of tumour-associated macrophages (M2) markers and M2 macrophages further enhanced HCC invasiveness.^3^ Injection of M2 macrophages could significantly promote tumour growth in orthotopic HCC-implanted mice, which further demonstrated the pro-tumoral role of M2 macrophage in HCC. Reprogramming of TAM away from M2-like, towards M1-like phenotype in tumour microenvironment by FDA-approved anti-HCC drug sorafenib suppressed hepatoma cell proliferation.^4^ These studies suggested the possibility of targeting TAMs as a potential immune-modulating strategy in HCC.

Recent studies increased attention on the plasticity of macrophage in disease progression and control. Under pathological and pharmacological conditions, macrophages acquire distinct phenotypic characteristics through different activation mechanisms. The classical activated macrophage (M1-like), exhibits pro-inflammatory properties by expressing and secreting pro-inflammatory molecules including TNF-*α*, IL6, IL12, IL1, Type I IFN*γ*, CXCL1–3, CXCL-5 and CXCL8–10; while macrophage could be alternatively activated to M2-like phenotype, which expresses anti-inflammatory factors such as IL10.^5^ M1-like macrophage, which strongly expresses CD86, iNOS and MHC-II, is distinguishable in phenotype from M2-like macrophage that has a higher level of CD206, CD163, YM1 and Fizz1.^6^ Tumour-associated macrophages (TAMs) are a population of macrophages in tumour microenvironment, which functions in promoting cancer cell proliferation and invasion, regulating tumour neovascularization as well as cytotoxic T-cell function.^7, 8^ TAMs are predominantly M2 phenotype and exhibits immunosuppressive function. M2 macrophages tend to secrete pro-tumoral cytokines that support tumour growth and suppress T-cell proliferation. Depending on the tumour environment, TAMs phenotype would be reprogrammed to M1-like that are characterised for its antigen-presenting property and pro-inflammatory function, favours tumour regression.^9, 10^

In particular, the role of nuclear factor-kappa B (NF-*κ*B) in TAMs is enormously studied in recent years. NF-*κ*B transcription factor is composed of five protein members including p100/p52, RelB, p105/p50, RelA and c-Rel.^11^ Formation and regulation of transcription activities of NF-*κ*B dimers could be achieved by both canonical and non-canonical pathways. Although NF-*κ*B activation is generally considered to trigger pro-inflammation, it is still not conclusive whether it is necessarily in regulating the polarisation of TAMs. In fact, the role of NF-*κ*B in TAMs skewing seems to be controversial across different studies. It was shown that NF-*κ*B activity was suppressed in TAMs co-cultured with ovarian cancer stem cells, which is responsible for the M2 macrophages skewing;^12^ while in ATP-binding cassette transporter G1 (ABCG1)-knockout TAMs, NF-*κ*B activity was increased and TAMs exhibited M1 phenotypes and direct toxicity to tumor cells.^13^ Devaraj and colleagues showed that C-reactive protein could repolarise M2 macrophages to M1 phenotype, but it fails to prime those M2 macrophages with inactive NF-*κ*B.^14^ Combination treatment of TGF-*β* inhibitor with TLR7 ligand on TAMs leads to M1 reprogramming and associated with obvious nuclear translocation of NF-*κ*B;^15^ TLR9 ligand combined with IL-10 receptor antibody and CCL16 may activate NF-*κ*B and redirect TAMs towards M1 phenotype.^16^ Treatments were also designed in targeting on NF-*κ*B in TAMs. A small molecule azithromycin inhibits TLR4-related NF-*κ*B activity, which leads to reduction of M1 and increase of M2 cytokines;^17^ TAMs challenged with zoledronic acid achieved M1 phenotypes with increase of NF-*κ*B-inducible M1 cytokines expression;^18^ miRNAs-containing exosomes derived from Epigallocatechin gallate-treated tumour cells could be absorbed by TAMs, and led to elicited NF-*κ*B-associated M1 skewing.^19^ But the role of NF-*κ*B in TAM polarisation is far more complicated and not just depends on the expression level of NF-*κ*B components. It was found that p50-knockout TAMs or TAMs with RNA interference against p50 normally produce M1 cytokines, and reduced expression of p50 may switch M2-like macrophages to M1 phenotype.^20, 21^ However, overexpression of p50 was also suggested to defect NF-*κ*B pathway and led to M2 phenotype in cancer.^22, 23^ It was also observed that activation of IKK*β*/NF-*κ*B is required for maintaining M2 phenotype.^24^ Prostate cancer cell-derived CCN3 induced NF-*κ*B activation in macrophages and skewed TAMs to M2 phenotypes;^25^ an *in vivo* NF-*κ*B decoy may cause TAMs polarisation to M2 phenotype.^26^ All the above studies revealed the controversy of NF-*κ*B activation in TAMs polarisation and it is somehow considered to be highly dependent on different tumour types and experimental approaches.^11^ Chan *et al.*^27^ even demonstrated that NF-*κ*B activity is responsible for both M1- and M2-cytokines expression in human cytomegalovirus-infected macrophages. The nature of stimulus and physiological content may determine the role of NF-*κ*B in TAMs polarisation.^23^

Baicalin is a natural flavonoid present in several medicinal plants including *Scutellaria baicalensis* Georgi. Few studies have revealed the anti-tumour action of baicalin with *in vitro* cellular HCC models.^28, 29^ It was also reported that baicalin may be an immunomodulatory agent by affecting Th17 cells^30^ and dendritic cells.^31^ It was highlighted that immune response may be boosted up by baicalin treatment via increased expressions of IFN*γ* and IL12, which are the essential factors for activating all types of acquired immune response.^32^ This prompts us to take an insight into the action and mechanism of baicalin for potential cancer therapy. We investigated the impact of baicalin on repolarisation of TAM and its role in mediating HCC inhibition, as well as elaborated the mechanism underlying baicalin-induced autophagy in regulating TAM repolarisation.

## Results

### Inhibition of orthotopic HCC growth by baicalin requires presence of macrophages

**Figure 1:**
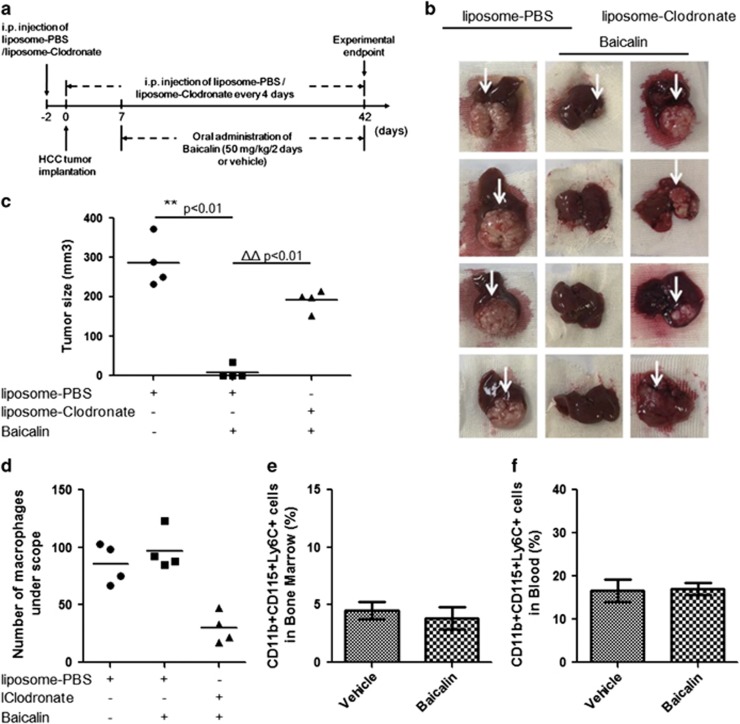
Inhibition of orthotopic HCC growth by baicalin requires presence of macrophages. (**a**) Schematic presentation of experimental design. (**b**) Removal of macrophage from mice completely blocked the anti-tumour effect of baicalin. Orthotopic HCC tumour growth in mice (*n*=4) with baicalin treatment was not obvious, whereas removal of macrophage by intraperitoneally injecting clodronate liposome resulted in relapse of hepatic tumour. (**c**) The size calculation of hepatic tumour. **P<0.01, compared with liposome control group without baicalin treatment; ^ΔΔ^*P*<0.01, compared with liposome control group with baicalin treatment. (**d**) The amount of intrahepatic tumour was slightly increased upon baicalin treatment without significance, while presence of clodronate liposome remarkably reduced hepatic macrophage. (**e**) Baicalin treatment did not potently induce production of inflammatory monocytes in bone marrow. (**f**) Baicalin treatment did not elevate inflammatory monocytes populations in circulating system

Few previous studies reported that baicalin may exhibit anti-tumour effect on HCC cells *in vitro*,^33, 34^ but investigation on the action of baicalin *in vivo* and its underlying mechanism remains scanty. To systematically evaluate the anti-tumour action of baicalin on HCC, we introduced an orthotopic HCC-implanted murine model by embedding small cubes of subcutaneously grown tumour onto the right lobe of mice liver, which has been reported in our previous studies,^33, 34, 35, 36^ followed by oral administration of baicalin (50 mg/kg) every alternate day after 1 week of transplantation. Body weight of mice was monitored once per week and there was no weight loss among the groups after 5 weeks of intervention (Supplementary Figure S1). Five-week treatment of baicalin could completely inhibit orthotopic HCC growth in mice, while relapse of tumour was observed in mice with macrophage removal (Figure 1b). The tumour size in macrophage-depleted mice with baicalin was not as large as that in macrophage-presenting mice without baicalin treatment; and significant relapse of tumour in macrophage-depleted mice in comparison to macrophage-presenting mice treated with baicalin revealed that macrophage is involved in mediating the anti-tumour effect of baicalin (Figure 1c). Immunohistochemistry staining with F4/80 antibody showed successful removal of macrophage by liposomal clodronate treatment, and baicalin did not significantly increase the residence of macrophage in liver. All these indicated that the role of macrophage in mediating anti-tumour effect of baicalin is independent to the increase of macrophages infiltration (Figure 1d). This was further evidenced by the observation that there was no significant increase in inflammatory monocyte in bone marrow or circulating system of baicalin-treated mice (Figures 1e and f). Our results reveal that macrophage has a positive role in regulating the anti-tumour effect of baicalin in HCC.

### Baicalin skews macrophages away from M2-like, towards M1-like phenotype

**Figure 2:**
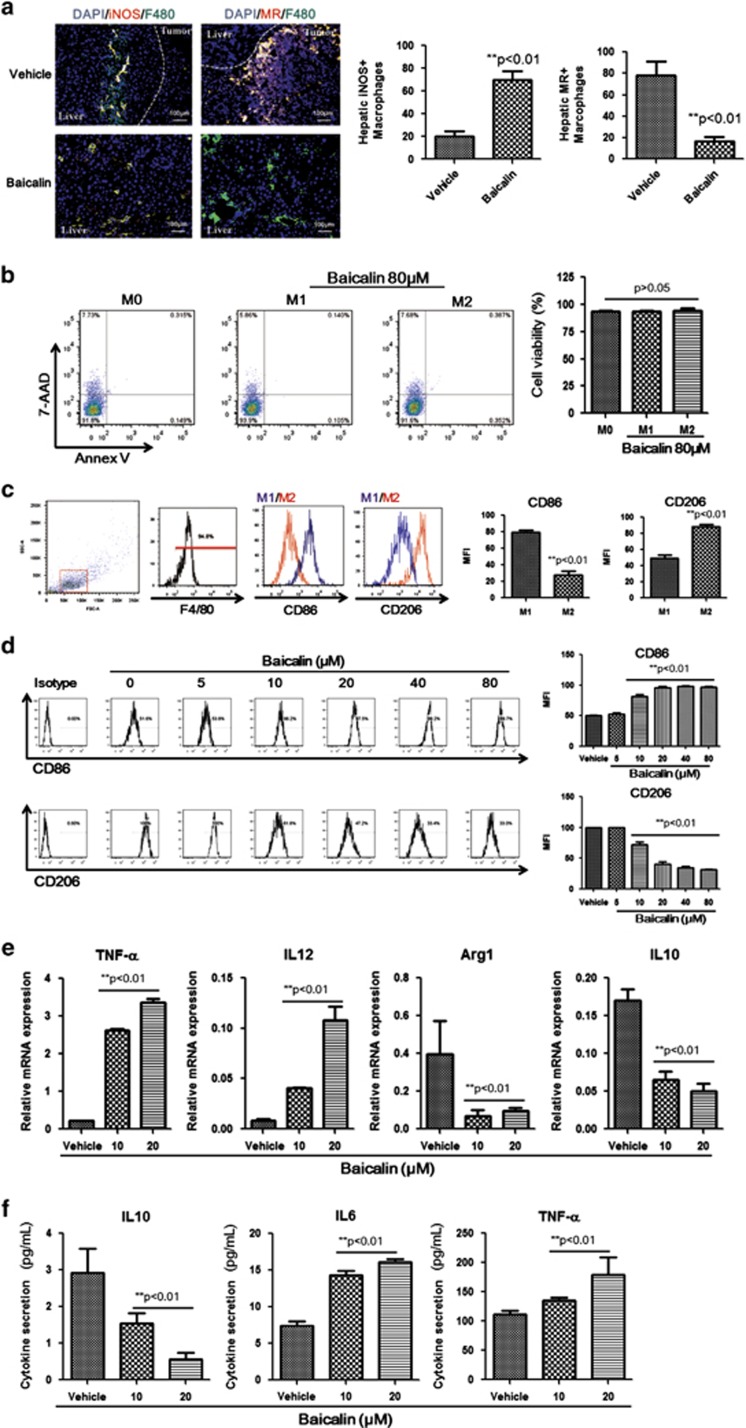
Treatment of baicalin results in reprogramming of M2-like TAMs to M1-like phenotype. (**a**) Baicalin treatment resulted in increase of hepatic M1-like TAMs with reduced M2-like populations. Antibodies against iNOS (M1-like marker) and mannose receptor (MR, M2-like marker) were used to stain frozen-sectioned liver tissue. Increase of iNOS-positive cells all over the liver of baicalin-treated mice was observed (*P*<0.01); while hepatic MR-positive cells within non-tumour and peri-tumour area was remarkably reduced upon baicalin treatment (*P*<0.01) (scale bar, 100 *μ*m); (**b**) baicalin did not cause cell death in any specific type of macrophages. BMDM was cultured and differentiated as described in Materials and Methods. A relatively high dose of baicalin (80 *μ*M) was given to either M1-like or M2-like TAM for 48 h. Cell viability was determined with flow cytometry using Annexin V/7-AAD staining. (**c**) Differentiated BMDM exhibited differential patterns in CD86/CD206 expression. Antibodies against CD86 and CD206 were used to stain cell surface marker of differentiated BMDMs. F4/80 was co-stained to identify differentiated macrophage population. M1-like macrophage had higher expression of CD86 with lower expression of CD206 (*P*<0.01), while CD206 was induced and CD86 was relatively low in M2-like macrophage (*P*<0.01). (**d**) Baicalin treatment resulted in skewing of TAM to M1-like macrophage. TAMs were induced by culturing BMDM with tumour supernatant (TSN) as described in Materials and Methods. Cells were subjected to different doses of baicalin for 48 h. It was observed that baicalin induced skewing of TAMs into M1-like phenotype and away from M2-like in dose-dependent manner. (**e**) Cytokine profile in baicalin-treated TAMs favouring M1-like phenotype. RNA from baicalin-treated cells was collected and expression of TNF-*α*, IL12 (M1-like macrophage markers) and IL10, Arginase1 (Arg1, M2-like macrophage markers) was determined by quantitative real-time PCR. Increase of M1 markers but decrease of M2 markers was observed in baicalin-treated TAM (*P*<0.01). (**f**) Baicalin treatment induced elevated secretion of pro-inflammatory cytokine in TAMs. Cytokine concentration in cultured TAM supernatant was determined with CBA kit. TNF-*α* and IL6 (pro-inflammatory cytokines) levels were increased (*P*<0.01), while IL10 (anti-inflammatory cytokine) was reduced after baicalin intervention (*P*<0.01). Cytokine level was expressed as pg/ml±S.D.

A plenty of studies have revealed that carcinogenesis involves a process of macrophage infiltration into tumour microenvironment followed by polarisation of macrophage by tumour-favouring factors.^37^ Infiltrated macrophages are programmed to anti-inflammatory M2-like phenotype, which protects tumour cells from immune surveillance.^38^ M1-like macrophages produce pro-inflammatory cytokines (TNF-*α*, IL12, iNOS, etc), which are against tumour cell growth, whereas M2-like phenotype secrets a series of tumour-favouring factors (IL10, TGF-*β*, Arginase1, etc) that promote tumour expansion.^39^ To examine if baicalin could re-programme intrahepatic macrophages towards M1-like phenotype, we measured the presence of M1/M2-like macrophages in the liver of baicalin-treated mice. Baicalin treatment increased the iNOS^+^ M1-like macrophages population while significant reduction of MR^+^ M2-like population in liver tissue of mice (Figure 2a). We also found that baicalin exhibited no specific cytotoxicity to M1/M2-like macrophages (Figure 2a), which indicated that the effect of baicalin on intrahepatic macrophages phenotype differentiation is independent to cell viability. To further classify the phenotype of baicalin-treated macrophages, we introduced two cell surface markers to distinguish M1-like phenotype from M2. M1-like macrophage possessed relatively higher expression of CD86 while M2-like macrophage highly expressed CD206 (Figure 2c). It was shown that after 7 days of differentiation with HCC tumour supernatant (TSN), the TSN-derived TAMs exhibited high expression of CD206 with relatively low CD86; indicating TAMs are prone to M2-like phenotype (Figure 2d). The 48-h treatment of baicalin re-skewed the TAMs away from M2-like, towards M1-like phenotype dose-dependently (Figure 2d). Consistent with the phenotypic analysis, we observed upregulation of the pro-inflammatory genes, TNF-*α* and IL12, while downregulation of IL10 and Arginase 1, the anti-inflammatory genes in baicalin-treated F4/80^+^ TAMs (Figure 2e). Baicalin-treated macrophage cells also secreted higher level of pro-inflammatory cytokines (IL6 and TNF-*α*) while lower level of IL10 (Figure 2f), which revealed TAM reprogramming by baicalin is functional in regulating cytokine production. Overall, baicalin promoted the TAM repolarisation towards M1-like phenotype.

### Baicalin induces repolarisation of M2-like macrophage without affecting M1 phenotype

**Figure 3:**
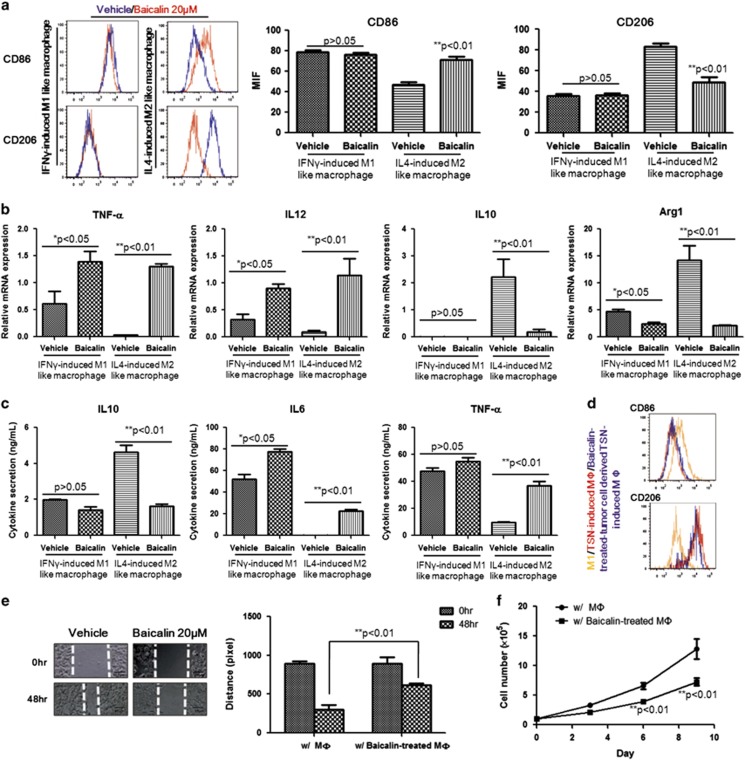
Baicalin induces repolarisation of M2-like macrophage without affecting M1 phenotype. (**a**) The 20 *μ*M baicalin treatment repolarised differentiated M2-like macrophage to M1 phenotype, while minimally affected differentiated M1-like macrophage. BMDMs were cultured and differentiated, then treated with baicalin for 48 h. Expression of CD86/CD206 was determined (*P*<0.01). Increase of CD86 expression with CD206 suppression was found in baicalin-treated M2-like macrophage, while baicalin had minimal effect on the expression of cell surface markers in M1-like macrophage (*P*>0.05). This observation was further evidenced by cytokine expression profile (**b**) and cytokine secretion (**c**). (**d**) Baicalin had minimal effect on macrophage-polarisation factors derived by tumour cells. TSN was collected from HCC cells treated with either vehicle or 20 *μ*M baicalin, and was used to differentiate BMDMs as described in Materials and Methods. No significant skewing of TAM could be observed. Differentiated M1 macrophages were used as a positive control. (**e**) Co-culturing of baicalin-pre-treated TAMs suppressed motility of HCC cells. HCC cells were seeded in 24-well cell culture plate and a gap was created by scraping with a 10-*μ*l pipette tip at the centre of the monolayer. Then HCC cells were subject to a 48-h co-culturing with TAM pre-treated with vehicle or 20 *μ*M baicalin for 48 h. HCC cells co-cultured with baicalin-pre-treated TAM exhibited slow migration towards centre of the gap (*P*<0.01). (**f**) Co-culturing of baicalin-pre-treated TAM inhibited proliferation of HCC cells. HCC cells (1 × 10^4^) were seeded and subjected to co-culturing with TAMs pre-treated with vehicle or 20 *μ*M baicalin. Number of HCC cells was counted at day 3, 6 and 9. Reduced proliferation of HCC cells with baicalin-pre-treated TAM was observed in time manner (*P*<0.01)

To further examine the action of baicalin on repolarisation of differentiated macrophages, we polarised BMDMs-derived macrophages to either M1-like or M2-like phenotype. The polarised macrophages were then subject to 48 h treatment of baicalin. Interestingly, baicalin particularly skewed M2-like macrophages towards M1-like phenotype, without causing any significant phenotypic changes in M1-like macrophage (Figure 3a). This was further evidenced by the fact that expression of pro-inflammatory genes in baicalin-treated M2-like macrophages was increased while anti-inflammatory factors were reduced (Figure 3b). In M1-like macrophages, expression of pro-inflammatory genes also increased though no phenotypic changes were observed after baicalin treatment, indicating that baicalin treatment could promote pro-inflammatory cytokines production. Consistent observation was obtained in culture supernatant of both M1-like and M2-like macrophages with baicalin treatment (Figure 3c). As M1-like TAMs may exert their anti-tumour action by producing pro-inflammatory cytokines,^40^ these observations revealed that baicalin treatment may result in polarised macrophages in tumour microenvironment prone to acquire anti-tumour functions. And the effect of baicalin on TAMs repolarisation in tumour microenvironment may be independent to any changes in polarisation factors derived by tumour cells itself, as phenotypic analysis showed that TAMs cultured with TSN from HCC cells with pre-treatment of baicalin were not prone to M1-like phenotype (Figure 3d). All the above observations confirmed the direct reprogramming action of baicalin on polarised TAMs. Furthermore, co-culturing of baicalin-pre-treated TAMs with HCC cells suppressed motility of cancer cells. HCC cells were seeded with 100% confluence in 24-well cell culture plate and a gap was created by scraping with a 10-μl pipette tip at the centre of the monolayer. Then HCC cells were subject to 48 h co-culturing with TAMs pre-treated with vehicle or 20 *μ*M baicalin for 48 h. HCC cells co-cultured with baicalin-pre-treated TAMs exhibited slow migration towards centre of the gap (Figure 3e, *P*<0.01). Also, co-culturing of baicalin-pre-treated TAMs inhibited proliferation of HCC cells. HCC cells (1 × 10^4^) were seeded and were subject to a co-culturing with TAMs pre-treated with vehicle or 20 *μ*M baicalin. Number of HCC cells was counted at day 3, 6 and 9. Reduced proliferation of HCC cells was observed in time-dependent manner (Figure 3f, *P*<0.01). Our results indicate that baicalin suppressed tumour cell expansion by incurring direct reprogramming effect on polarised macrophages.

### Induction of autophagy is involved in repolarisation of baicalin-treated TAMs

**Figure 4:**
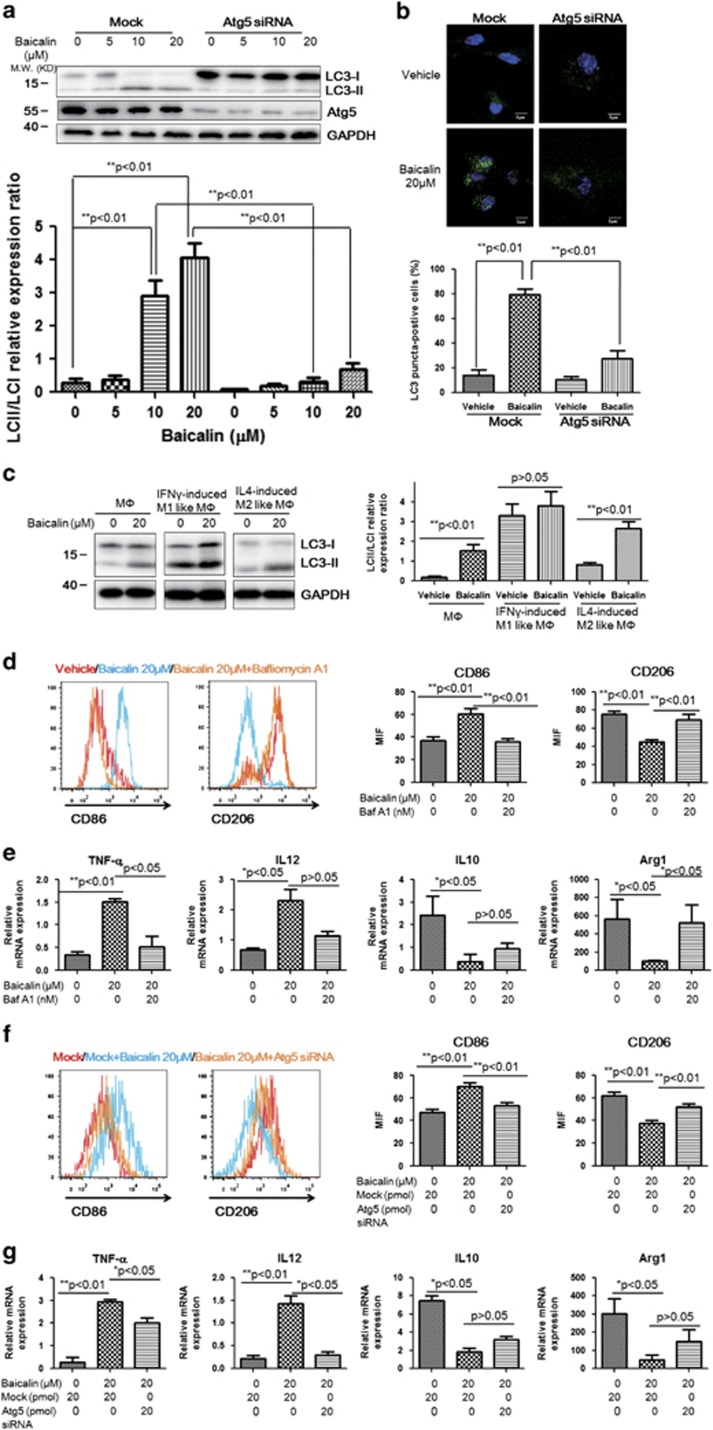
Autophagy is involved in baicalin-induced TAM repolarisation. (**a**) Baicalin treatment induced conversion of LC3-I into LC-II in TAM. BMDM cells were subjected to RNA interference as described in Materials and Methods with mock scramble negative control or siRNA against murine Atg5. Cells were treated with baicalin for 48 h and protein was collected for analysis. Increase of LC3-II expression with reduced LC3-I upon baicalin treatment was observed, while suppression of Atg5 attenuated the conversion of LC3-I into LC-II. (**b**) Baicalin increased LC3 puncta in TAM. Cells with mock or Atg5 RNA interference were subjected to baicalin treatment. Then cells were stained with LC3B antibody and visualised under confocal microscope. Increase of LC3 puncta (light green dots) was observed in baicalin-treated TAM, which could be blocked by RNA interference against Atg5 (magnification: x60). (**c**) Baicalin could induce autophagy in M2-like macrophage but not in M1-like. Protein expression was detected with immunoblotting. Conversion of LC3-I into LC3-II was observed in both unpolarised macrophage and M2-like macrophage, but not in M1-like macrophage. (**d**) Blockade of autophagy by presence of Bafilomycin A1 resulted in re-skewing of baicalin-treated TAMs into M2 phenotype. Bafilomycin A1 was added to TAM 30 min before the 48-h baicalin treatment. It was observed that presence of Bafilomycin A1 potently blocked change of CD86 and CD206 expression induced by baicalin. This was further evidenced by observations in cytokine expression profile (**e**) of baicalin-treated TAMs with or without Bafilomycin A1. (**f**) Suppression of autophagy by RNA interference against Atg5 induced re-skewing of baicalin-treated TAM to M2 phenotype. RNA interference was conducted as described and TAMs was subject to baicalin treatment. Suppression of Atg5 expression significantly blocked change of CD86 and CD206 expressions induced by baicalin. This was further evidenced by observations in cytokine expression profile (**g**) of baicalin-treated TAM with or without RNA interference against Atg5

Some previous studies have reported the property of baicalin as a natural autophagy inducer in cancer cells,^34, 41^ and consistently, we observed increased of LC3-II/LC3-I ratio in baicalin-treated TAMs. The conversion of cytoplasmic form of LC3 into membrane form in baicalin-treated TAMs indicated induction of autophagy (Figure 4a). We also observed autophagy induction in TAMs on liver tissue of HCC bearing mice (Supplementary Figure 7). Elevated LC3 punctuation in baicalin-treated TAMs further proved the autophagy initiation, and baicalin-induced autophagy could be blocked by RNA interference against autophagy essential gene Atg5 (Figure 4b). It was found that induction of autophagy by baicalin could only be achieved in unpolarised and M2-like macrophages but not in M1-like macrophages (Figure 4c). Considering that,^42^ this finding may indicate autophagy plays a role in baicalin-induced macrophage polarisation. To prove this hypothesis, we used Bafilomycin A1 and RNA interference against Atg5 gene to suppress induction of autophagy in TAMs. Pharmacological inhibition of autophagy by Bafilomycin A1 abolished repolarisation of TAM by baicalin towards M1-like phenotype (Figure 4d), and it was further confirmed by reduced pro-inflammatory genes and elevated anti-inflammatory genes in baicalin-treated TAMs in the presence of Bafilomycin A1 (Figure 4e). Consistent observations was obtained on reprogramming of baicalin-treated TAMs towards M2-like phenotype upon Atg5 depletion, and reverted changes of relative gene expression induced by baicalin (Figures 4f and g). These observations confirmed autophagy is involved in reprogramming of TAM induced by baicalin.

### Autophagy-associated activation of RelB/p52 pathway mediates repolarisation of TAM by baicalin

**Figure 5:**
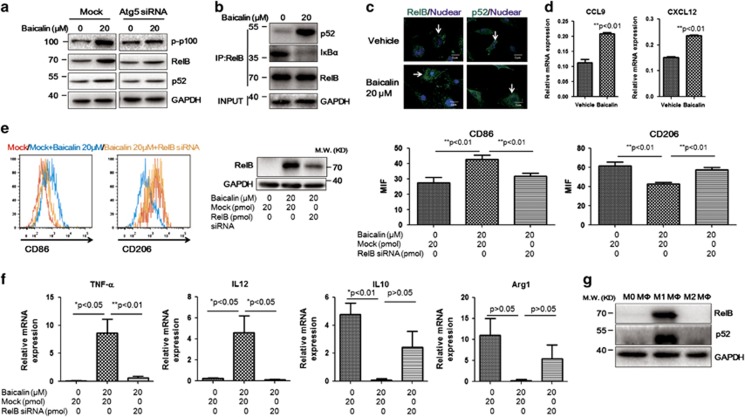
Autophagy-associated activation of RelB/p52 pathway mediates TAM repolarisation induced by baicalin. (**a**) The induced expression of RelB in baicalin-treated TAM is associated with autophagy induction. RNA interference against Atg5 was conducted as described and TAM was treated with 20 *μ*M baicalin for 48 h. Expression of RelB, p-p100 and p52 was detected by immunoblotting. It was shown that baicalin treatment resulted in increase of RelB and p52 expression, as well as phosphorylation of p100, and this effect of baicalin could be blocked by inhibition of autophagy by RNA interference against Atg5. (**b**) Baicalin treatment induced association of p52 with RelB. TAM was treated and co-immunoprecipitation assay was conducted as described. Increased association of RelB with p52 was observed in baicalin-treated TAM. Reduced I*κ*B*α* with RelB was also observed. Expression of protein level was normalised by GAPDH as INPUT samples and by RelB as IPed-samples. (**c**) Baicalin treatment induced nuclear localisation of RelB and p52. DAPI was used to stain nuclei of the cells. TAM with or without baicalin treatment was fixed and stained with antibodies against RelB and p52, followed by observation under confocal microscope. Increased RelB and p52 presentation in nuclear area was found in baicalin-treated cells. (**d**) Elevation of RelB/p52-specific target gene expression CCL19 and CXCL12 was observed in TAM in the presence of baicalin (magnification: x60). (**e**) Suppression of RelB by RNA interference induced re-skewing of baicalin-treated TAMs to M2 phenotype. RNA interference was conducted as described and TAM was subjected to baicalin treatment. Suppression of RelB expression significantly blocked change of CD86 and CD206 expression induced by baicalin. This was further evidenced by observation in cytokine expression profile (**f**) of baicalin-treated TAMs with or without RNA interference against RelB. (**g**) Expression of RelB and p52 in different phenotypes of macrophages. BMDMs were cultured and differentiated as described and protein was collected for analysis. It was observed that RelB and p52 were highly expressed in M1-like macrophage but not in either unpolarised or M2-like macrophage

To explore the mechanism underlying autophagy-induced repolarisation of TAM by baicalin, we examined the activation of RelB/p52 pathway, which was reported to dominate reprogramming of Th1 cells to Th2 phenotype.^43^ Increased RelB expression was observed in baicalin-treated TAMs, which could be attenuated by depletion of Atg5 (Figure 5a). Interestingly, it was observed that in baicalin-treated TAMs, the mRNA expression of RelB was elevated (Supplementary Figure S2). The consistent increase of phosphorylation of p100 and p52 in baicalin-treated TAMs revealed that the transcriptional increase of RelB may be due to the positive feedback loop of RelB/p52 activation.^44^ Co-immunoprecipitation assay with RelB antibody confirmed association of p52 with RelB in TAMs, while I*κ*B*α* was dissociated from the RelB/p52 complex with baicalin treatment (Figure 5b). Furthermore, it was observed that RelB and p52 were translocated into nuclear area of TAMs in the presence of baicalin (Figure 5c), and transactivated the RelB/p52-specific target genes CCL9 and CXCL12 (Figure 5d). Inhibition of RelB expression by RNA interference in baicalin-treated TAMs abolished repolarisation of TAMs towards M1-like phenotype, and drove the tendency of TAMs towards M2-like phenotype. Increased expression of pro-inflammatory cytokines by baicalin was abolished by RelB suppression, and reduction of anti-inflammatory cytokines was restored (Figure 5f). The high expression of RelB and p52 in M1-like macrophages instead of in unpolarised and M2-like macrophages (Figure5g) further confirmed the role of upregulated RelB in mediating repolarisation of TAM induced by baicalin.

### Activation of RelB/p52 pathway may be dependent on reduced expression of TRAF2 in baicalin-treated TAMs

**Figure 6:**
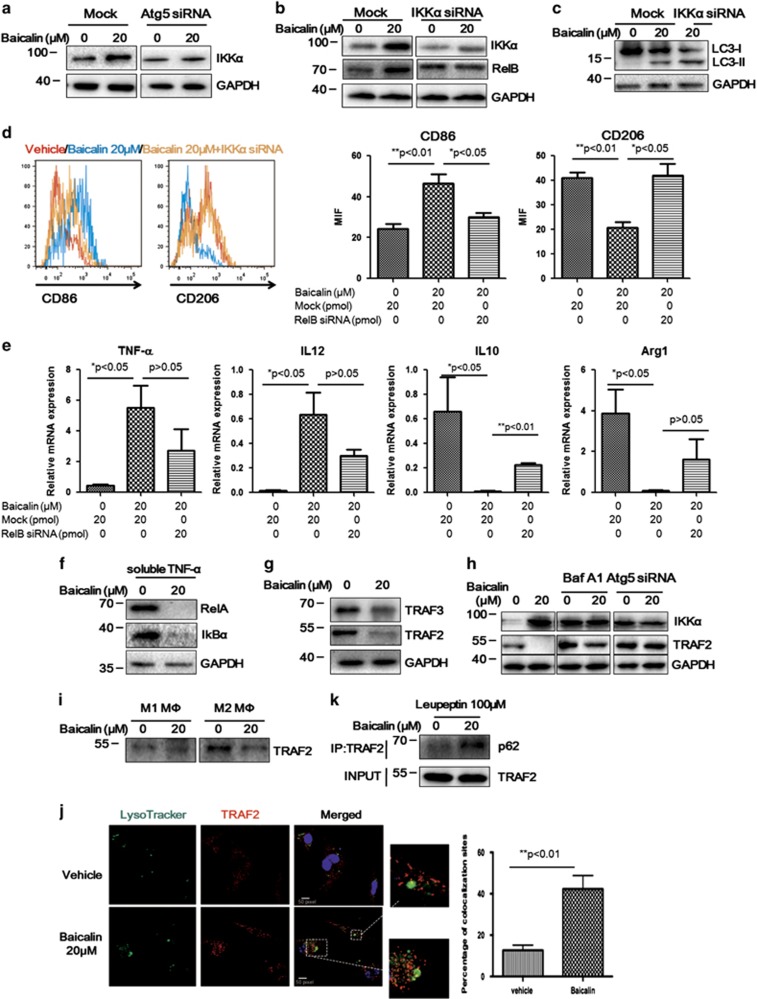
Autophagy-induced TRAF2 degradation may be associated with the activation of RelB/p52 pathway in baicalin-treated TAMs. (**a**) Induced expression of IKK*α* in baicalin-treated TAMs is associated with autophagy induction. RNA interference against Atg5 was conducted as described and TAM was treated with 20 *μ*M baicalin for 48 h. Expression of IKK*α* was detected by immunoblotting. It was shown that baicalin treatment resulted in increase of IKK*α* expression, which could be blocked by inhibition of autophagy by RNA interference against Atg5. (**b**) Silencing of IKK*α* expression attenuated RelB overexpression in baicalin-treated TAM. RNA interference against IKK*α* was conducted as described and TAM was treated with 20 *μ*M baicalin for 48 h. It was shown that increased expression of RelB by baicalin was attenuated upon IKK*α* suppression. (**c**) Autophagy induction is independent to IKK*α* overexpression. (**d**) Suppression of IKK*α* by RNA interference induced re-skewing of baicalin-treated TAM to M2 phenotype. RNA interference was conducted as described and TAM was subjected to baicalin treatment. Suppression of IKK*α* expression significantly blocked change of CD86 and CD206 expression induced by baicalin. This was further evidenced by observation in cytokine expression profile (**e**) of baicalin-treated TAMs with or without RNA interference against IKK*α*. (**f**) RelA pathway may not be activated upon baicalin treatment. TAM was treated with baicalin in the presence of soluble TNF-*α*. Expression of RelA and I*κ*B*α* was determined. (**g**) Baicalin treatment induced reduction of TRAF2 and TRAF3 expression. (**h**) Baicalin-suppressed TRAF2 expression may be blocked when autophagy was blocked by either Bafilomycin A1 or RNA interference against Atg5. (**i**) Baicalin induced TRAF2 degradation in M2-like macrophage but not in M1 phenotype. (**j**) Degradation of TRAF2 induced by baicalin may be dependent on autophagy-associated lysosomal pathway. TAM treated with or without baicalin was fixed and stained with TRAF2 antibody. Lysosome was stained with Lysotracker Green for 30 min. It was observed that TRAF2 was translocated to lysosome after treatment of baicalin in TAM (magnification: x60). (**k**) p62 cargo protein may dominate selective autophagic degradation of TRAF2 in baicalin-treated TAM. TAM was treated with or without baicalin in the presence of lysosome inhibitor leupeptin. Co-immunoprecipitation assay was conducted with TRAF2 antibody and association of p62 with TRAF2 was detected by immunoblotting. Recruitment of p62 to TRAF2 upon baicalin treatment was observed

To further prove the mechanism, we observed the expression of IKK*α*, the upstream event of RelB/p52. A consistent upregulation of IKK*α* was observed in baicalin-treated TAMs, which was blocked by inhibition of Atg5 (Figure 6a). Similar to RelB, it was found that expression of IKK*α* mRNA was elevated (Supplementary Figure S3), majorly due to the positive feedback regulation of RelB/p52 activation.^45^ The role of IKK*α* in mediating baicalin-induced RelB activation was further proved by the observation that IKK*α* inhibition by RNA interference attenuated upregulation of RelB expression (Figure 6b). Interestingly, it was shown that blockade of IKK*α* has minimal effect on baicalin-induced autophagy in TAM, indicating that IKK*α* positions at the downstream of autophagy (Figure 6c). Silencing of IKK*α* in baicalin-treated TAMs restored M2-like phenotypic characteristics of the cells (Figure 6d), and suppressed pro-inflammatory gene expression in baicalin-treated TAMs, as well as restored the expression of anti-inflammatory genes (Figure 6e). As IKK*α* also plays a key role in mediating activation of RelA by inducing phosphorylated-degradation of I*κ*B*α*,^46^ which then activate transcription of pro-inflammatory genes, we examined if RelA pathway was affected by baicalin treatment in TAMs. Interestingly, we observed very low expression level of RelA in TAMs, and baicalin treatment could suppress RelA even in the presence of soluble TNF-*α*, a RelA-activating cytokine (Figure 6f). This was consistent with the previous report that TLR2 could selectively suppress RelA expression upon induction of autophagy.^47^ This data revealed that repolarisation of TAM by macrophage may be dependent on activation of RelB but not RelA pathway. In this case, we then examined the expression of TRAF2/TRAF3 complex, the negative regulator of RelB pathway, and observed the reduced expressions of both TRAF2 and TRAF3 (Figure 6g). Our data may suggest restriction of TRAF2/TRAF3 expression, the intracellular suppressor of IKK*α*/RelB/p52 signalling, may confer repolarisation of TAM by baicalin.

### Baicalin initiates autophagic degradation of TRAF2 in TAMs

In our study, we did not observe reduced mRNA expression of TRAF2 in baicalin-treated TAM (Supplementary Figure S4), though protein level of TRAF2 was potently suppressed (Figure 6g). This inhibitory effect of baicalin on TRAF2 was completely abolished in the presence of pharmacological inhibitor of autophagy Bafilomycin A1, or by depleting Atg5 from TAMs (Figure 6h), indicating that reduced expression of TRAF2 in baicalin-treated TAMs may be independent to transcriptional suppression of TRAF2 itself but dependent on autophagic degradation pathway. And this effect of baicalin was specific to M2-like macrophages, as expression of TRAF2 was relatively low in M1-like macrophages and baicalin showed minimal effect on it (Figure 6i). This selective autophagic degradation of TRAF2 in baicalin-treated TAMs was further proved by observation of co-localization of TRAF2 with lysosome and monodansylcadaverine (MDC), indicating a lysosomal degeneration of TRAF2 induced by baicalin (Figure 6j and Supplementary Figure S6). Co-immunoprecipitation assay with LC3 and TRAF2 antibody also confirmed the association of TRAF2 with LC3 and Atg5 in TAMs after baicalin intervention (Supplementary Figure S6). Furthermore, a cargo protein of selectively autophagic degradation, p62, was observed to attach TRAF2 upon baicalin treatment (Figure 6k). These observations reveal a lysosomal degradation of TRAF2 is dependent on baicalin-induced autophagy in TAMs.

## Discussions

Macrophages are high-plasticity immune cells, and they maintain in M1/M2 mixture states under normal condition. Macrophage M1/M2 state switching may be crucial in determining the tissue fate.^48^ High expression of M-CSF and macrophages markers in peritumoral area of hepatocellular carcinoma tissue promotes tumour recurrence and metastasis; distribution of M2 macrophages in peritumoral section is associated with poor prognosis of HCC patients.^3, 49^ Depletion of TAMs further improves the anti-tumour effect of sorafenib, the current first-line treatment of HCC.^50^ Therapeutics against M2 macrophage is no doubt, in most of the studies, benefits cancer therapy. However, the role of M1-like macrophage is much more complicated as previous studies showed that the inflammatory environment conditioned by macrophages promoted tumorigenesis and cancer progression. It was shown that IL6-producing TAM may promote the expansion of cancer stem cell in HCC.^51^ Although phenotype of TAM cannot be identified here, as both M1-like and M2-like macrophages could produce IL6,^52^ it indicates the role of pro-inflammatory M1 macrophages may not be as direct as M2 macrophages. A recent study observed that though TAM was repolarised towards M1 phenotype and away from M2 phenotype by sorafenib, the circulating inflammation condition was reduced by the treatment.^4^ This may indicate that skewing of M2-like TAM to M1 phenotype will not promote systematic inflammation in cancer patients. In fact, HCC carcinogenesis would request infiltration of inflammatory leukocytes into the liver, which may cause hepatocyte apoptosis and sustained inflammation.^53^ Our study, showing that baicalin did not elevate circulating inflammatory monocytes, revealed that the compound may be able to repolarise loco-regional TAM away from M2 phenotypes. In fact, in our co-culture study, we did not observe significant apoptosis of HCC cells in the presence of baicalin-pre-treated TAMs (data not shown), which may indicate that production of pro-apoptotic factors by baicalin-treated TAMs, such as TNF-*α*, is not sufficient to cause death of HCC cells. Our findings, though baicalin-treated TAMs are prone to be M1-like, we cannot preclude that reduced HCC cell proliferation and migration is not due to the decrease of M2-like TAM-derived protumoral cytokines.

**Figure 7:**
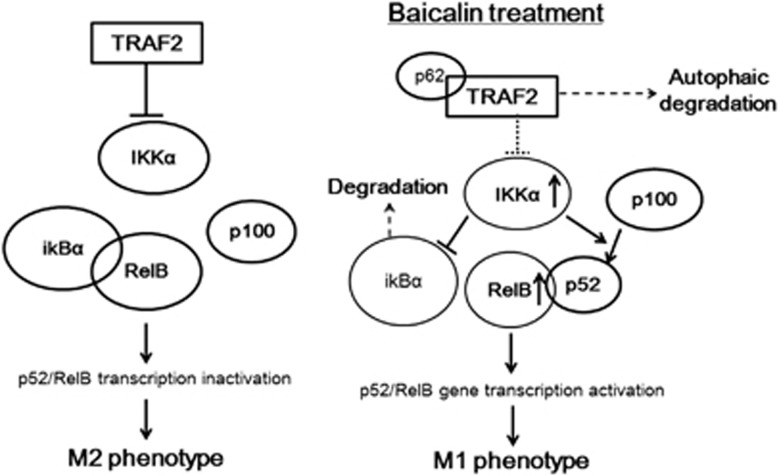
The mechanism underlying repolarisation of baicalin-treated TAMs to M1 phenotype

Furthermore, our study demonstrated that baicalin-mediated M1 reprogramming is regulated by the downregulation of TRAF2 via autophagy-dependent pathway. Detailed regulatory mechanism is illustrated in Figure 7. TRAF2 is a signalling protein in response to TNF receptor binding activity, and its regulation largely affected the downstream NF-*к*B activation. In TNF–RII complex, association of TRAF2 with c-IAP1 promotes TRAF2 ubiquitination-dependent degradation.^54^ The c-IAP1-mediated TRAF2 degradation is required for monocyte differentiation to functional macrophages.^55^ It is surprising that we found the blockade of autophagy also inhibited TRAF2 reduction, suggesting TRAF2 may be degraded in lysosome apart from the conventional proteasomal-related pathway. Although this scenario may be rare; the notion is supported by a recent study on TRAF2 lysosomal-dependent degradation mediated by zinc finger protein A20.^56^ We indeed observed the ubiquitination of TRAF2 (Supplementary Figure S5) and colocalization of TRAF2 with lysosomal tracker p62, the autophagy-related protein that cargo ubiquitinated intracellular molecules, which was recruited to autophagosome during autophagy process and lead to the targeted molecules degraded through lysosomal pathway.^57^ The interaction between ubiquitinated TRAF2 and p62 may explain TRAF2-lysosomal degradation is autophagy dependent. All these collectively suggest the recruitment of ubiquitinated TRAF2 to lysosomal compartments for clearance.

In our study, baicalin-mediated downregulation of TRAF2 promotes sustained activation of IKK*α* and RelB. IKK*α* is a tumour promoter in the context of colorectal cancer that is responsible in blocking the recruitment of M1-like myeloid cells.^58^ IKK*α* and RelB are important regulators of non-canonical NF-*к*B signalling and p100 processing contributes to the activation of RelB/p52 complex, while TRAF2 negatively regulates the pathway.^59^ Inactive form of RelB is associated with I*к*B*α* and it mobilises to nucleus followed by I*к*B*α* degradation.^60^ RelB is associated with p52 to activate transcription of target genes. This non-canonical activation of NF-*к*B pathway has been reported to dominate the reprogramming of Th1 cells into Th2 phenotype in cells other than macrophage.^43^ Besides, study has showed high nuclear p50 concentration in TAMs associated with M2-like phenotype, while TAMs isolated from p50-knockout mice expressed M1-like cytokines.^21^ The inhibitory effect of p50 on M1-reprogramming may be due to the suppression of NF-*κ*B transcriptional activities through formation of p50 homodimers.^61^ As p50 lacks of transactivation domain, p50/p50 homodimer is supposed to compete with p65/p50 heterodimer, which is able to activate transcription of pro-inflammatory genes.^11^ In fact, p52, the non-canonical NF-*κ*B pathway member, which also lacks of transactivation domain, is able to bind to itself or with p50 to act as a repressor of NF-*κ*B activation.^62^ These evidences showed that activation of NF-*κ*B pathway does not merely depend on increased expression of p50 or p52. It was addressed that the binding of NF-*κ*B dimers is highly adaptive to cellular situation. As the transactivation of pro-inflammatory genes by NF-*κ*B dimers is transient and under dynamic equilibrium, the nature of dimers are dependent on the given cellular concentrations of NF-*κ*B components.^63^ In our study, we found that RelB is increased upon baicalin treatment. Increase of RelB may in turn results in conversion of repressive p52 dimers to RelB/p52 heterodimers, which are able to transactive M1 genes expression. This was evidenced by previous study that challenging macrophages with LPS induced RelB expression, which in consequence increased transcription of non-canonical NF-*κ*B-targeted gene ELC.^62^ Also, this was consistent with previous observation that production of RelB is a major contributing factor in activation of non-canonical NF-*κ*B pathways.^64^ Study also revealed that removal of RelA by autophagy, the core molecule of canonical NF-*к*B pathway, may lead to M2 polarisation of macrophage,^47^ though there is still no direct evidence to show any essential role of RelA pathway activation in mediating macrophage reprogramming. This is consistent with our observation that baicalin-induced autophagy also resulted in RelA degradation in TAM. However, as baicalin-induced autophagy particularly promotes TRAF2 degradation-associated activation of RelB/p52 pathway; the effect of RelA degradation on TAM polarity seems to be compensated. In addition, it was found that production of pro-inflammatory stimuli particularly results in RelB activation but independent to RelA pathway in macrophage,^62^ which is consistent with our observation that RelB and p52 highly expressed in M1-like but not M2-like macrophage. A positive feedback loop in RelB pathway in baicalin-treated TAM resembles classical activation of macrophages into M1-like phenotype.

In conclusion, our data postulate that the tumour suppressive effect of baicalin was mediated by re-education of TAMs away from M2-like, towards tumour inhibiting M1-like phenotype. This effect was regulated by activation of RelB/p52 pathway via TRAF2 lysosomal degradation-dependent pathway. Baicalin-induced autophagy was responsible for lysosomal degradation of TRAF2 as well as TAM repolarisation. This study proposes baicalin as a potential immune therapeutic candidate for the treatment of hepatocellular carcinoma.

## Materials and Methods

### Chemical, reagents and antibodies

Baicalin, Bafilomycin A1 and leupeptin were purchased from Sigma-Aldrich (St. Louis, MO, USA). Antibody against MR was obtained from Abcam (Cambridge, UK); antibody against CD11b, Ly6C, CD115, F4/80, CD86 and CD206 were from eBioscience (CA, USA); antibodies against LC3B and iNOS was purchased from Novus Biologicals (Littleton, CO, USA); Antibodies against phosphor-p100, RelA, IKK*α*, RelB, TRAF2, TRAF3 IкB*α*, GADPH, were purchased from Cell Signalling Technology (MA, USA); The FITC-conjugated secondary antibody and LysoTracker Green were from Molecular Probe (Eugene, OR, USA), murine recombinant proteins M-CSF, IFN*γ* and IL4 were from Peprotech (NJ, USA).

### Cells, orthotopic HCC implantation animal model and macrophage removal

*In vitro* and *in vivo* models of interaction between HCC cells have been established and used in our previous studies.^36^ HCC cell line MHCC97L cells were provided by Professor Man Kwan in Department of Surgery, University of Hong Kong, and were maintained in DMEM supplemented with 10% fetal bovine serum. Murine orthotopic HCC model was established on BALB/cAnN-nu athymic mice. Removal of macrophage from mice was conducted as indicated in Figure 1a. In brief, 2 days before the implantation of HCC generated by MHCC97L cells, mice in macrophage-removal group (MΦ−) was given clodronate liposome via intraperitoneal injection once (0.1 ml/10 g, ClodronateLiposomes.com, the Netherlands) to deplete macrophage from mice. Macrophage removal was then conducted throughout the experiment by liposomal clodronate treatment every 4 days. Mice in macrophage-presenting groups (MΦ+) received equal volume of PBS liposome as control. A small cube (~1 mm^3^) of HCC tumour was implanted into the left lobe of mice liver. Presence of liver tumour was checked 1 week after implantation by laparotomy. Mice in macrophage-presenting groups were then given baicalin (50 mg/kg per 2 days) or equal volume of PBS (*n*=4). All mice were sacrificed after 5 weeks of treatment. The study protocols were approved by the Committee on the Use of Live Animals in Teaching and Research (CULATR) of The University of Hong Kong, Hong Kong (reference number: 2809-12). Size of the tumour (mm^3^) was measured as (length × width)^2^/2. The myeloid cells from bone marrow and circulating system were isolated for further flow cytometry analysis.

### Statistical analysis

Studies were performed in triplicate except particular notice in figures. Data were expressed as mean±S.D. Statistical significance were analysed using one-way ANOVA followed by Newman–Keuls *post hoc* test, and *P*-value<0.05 considered as significant.

Other Materials and Methods were present in Supplementary Methods.

## Abbreviations

TAM: tumour-associated macrophages
HCC: hepatocellular carcinoma
BMDM: bone marrow-derived monocytes
MR: mannose receptor
iNOS: inducible nitric oxide synthase
TNF-*α*: tumour necrosis factor-*α*
IL12: interleukin-12
IL10: interleukin-10
TGF-*β*: tumour growth factor-*β*
TSN: tumour supernatant
LC3: microtubule-associated protein 1 A/1B-light chain 3
Atg5: autophagy protein 5
TRAF: TNF receptor-associated factor
NF-*κ*B: nuclear factor kappa-B
