# Supplementary material for: Autophagy-induced RelB/p52 activation mediates tumour-associated macrophage repolarisation and suppression of hepatocellular carcinoma by natural compound baicalin
Source: Cell Death Dis. 2015 Oct 22;6(10):e1942–. doi: 10.1038/cddis.2015.271 (PMC4632300; doi:10.1038/cddis.2015.271)
Supplement: Supplementary Information [file cddis2015271x1.docx]

**Supplementary data**

Figure S1 Examination of body weight of mice

Figure S2 Baicalin induced mRNA expression of RelB in TAM

Figure S3 Baicalin elevated mRNA expression of IKKα in TAM

Figure S4 Baicalin has minimal effect on mRNA level of TRAF2 in TAM

Figure S5 TRAF2 was ubiquitinated in baicalin-treated TAM.

Figure S6 TRAF2 was associated with autophagosome.

Figure S7 RelB and LC3 expressions were increased in *in vivo* liver tissue of HCC bearing mice.

**Fig.S1 Examination of body weight of mice**


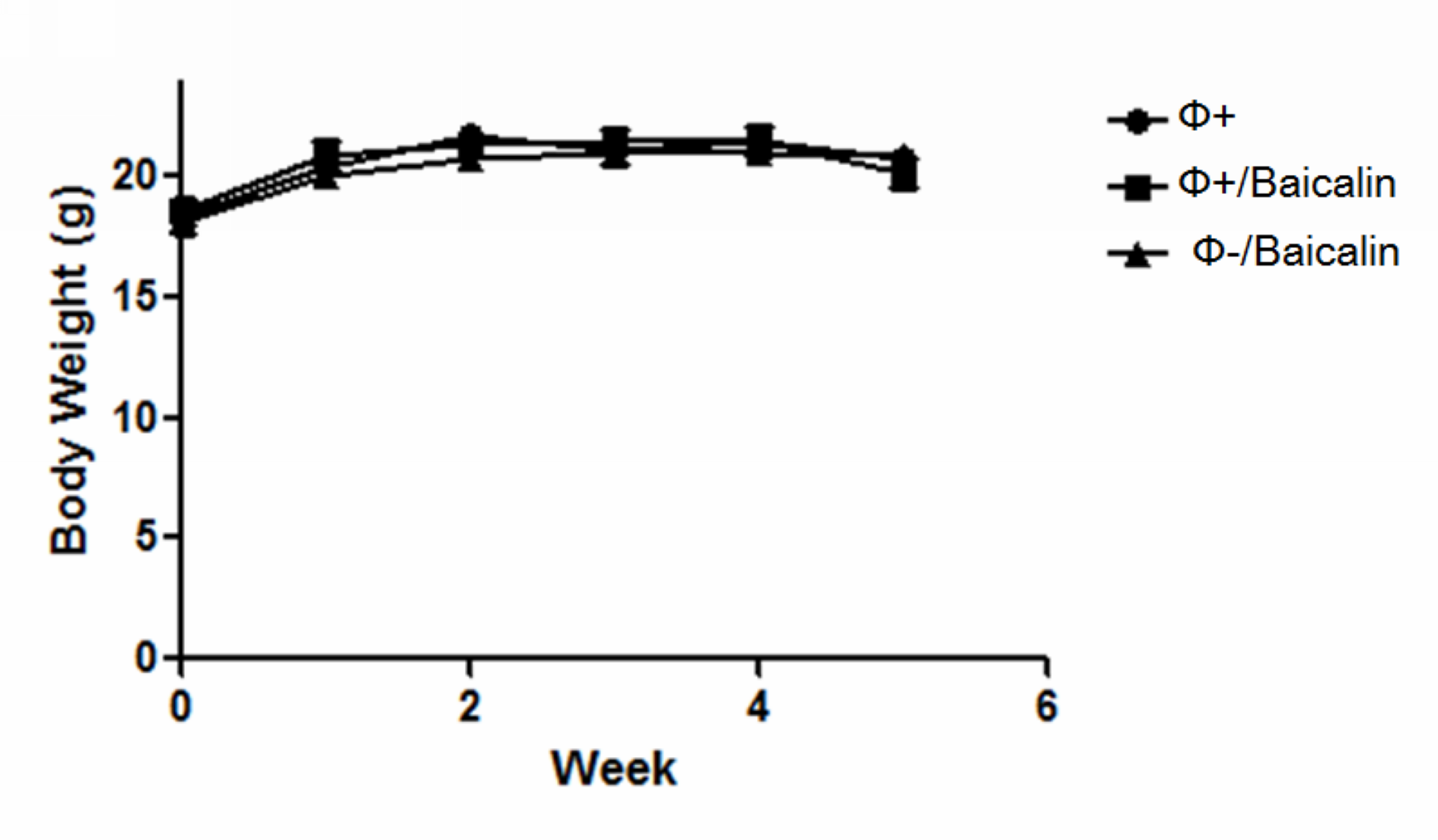


Animal model was established as described in materials and methods. Body weight of mice was measured every week. There are no significant differences in body weight across the groups.

**Fig. S2 Baicalin induced mRNA expression of RelB in TAM**


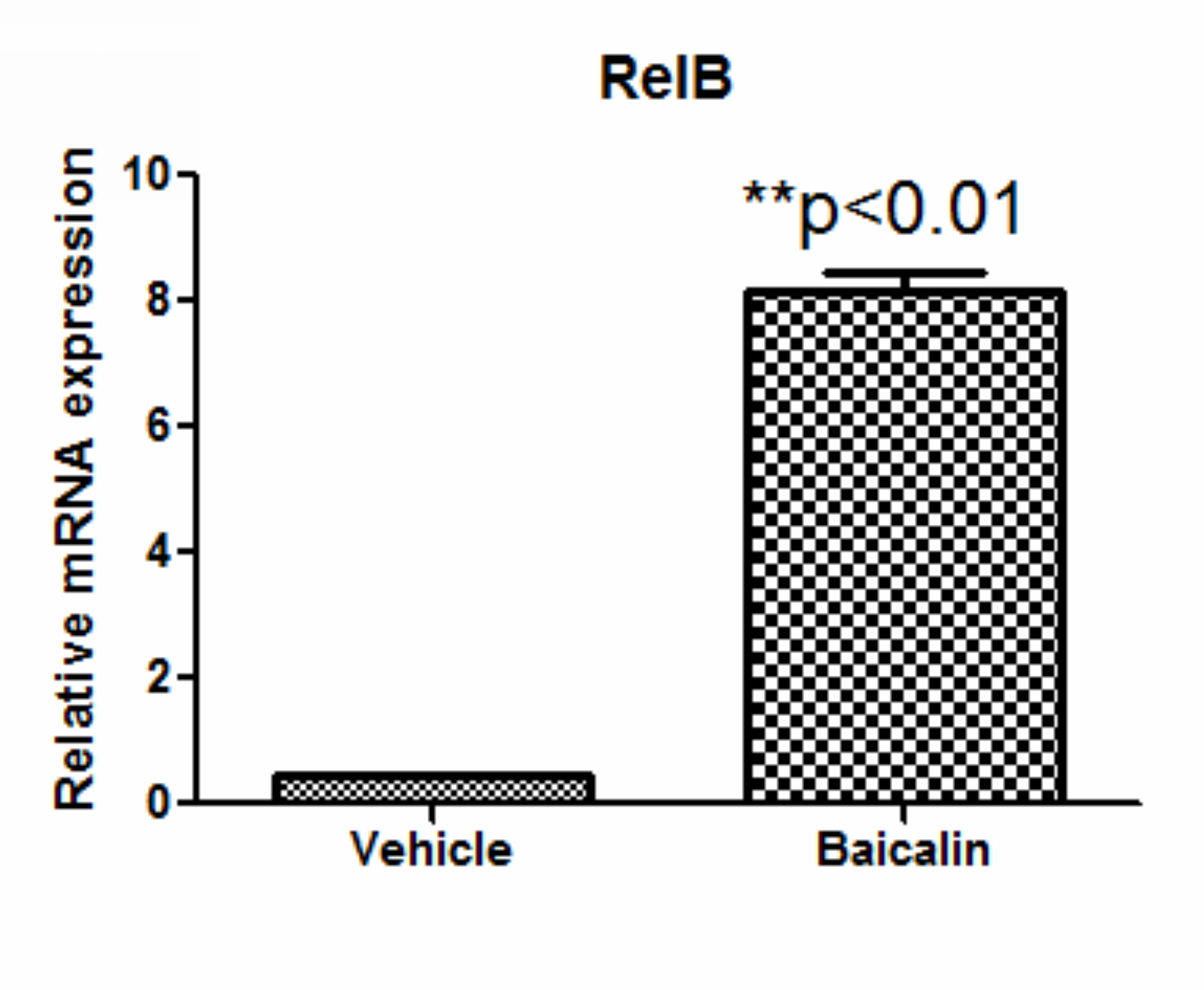


TAM was treated with vehicle or 20μM baicalin for 48 hours and mRNA expression of RelB was measured by qPCR. Significant increase of RelB mRNA in baicalin-treated TAM was observed.

**Fig. S3 Baicalin induced mRNA expression of IKKα in TAM**


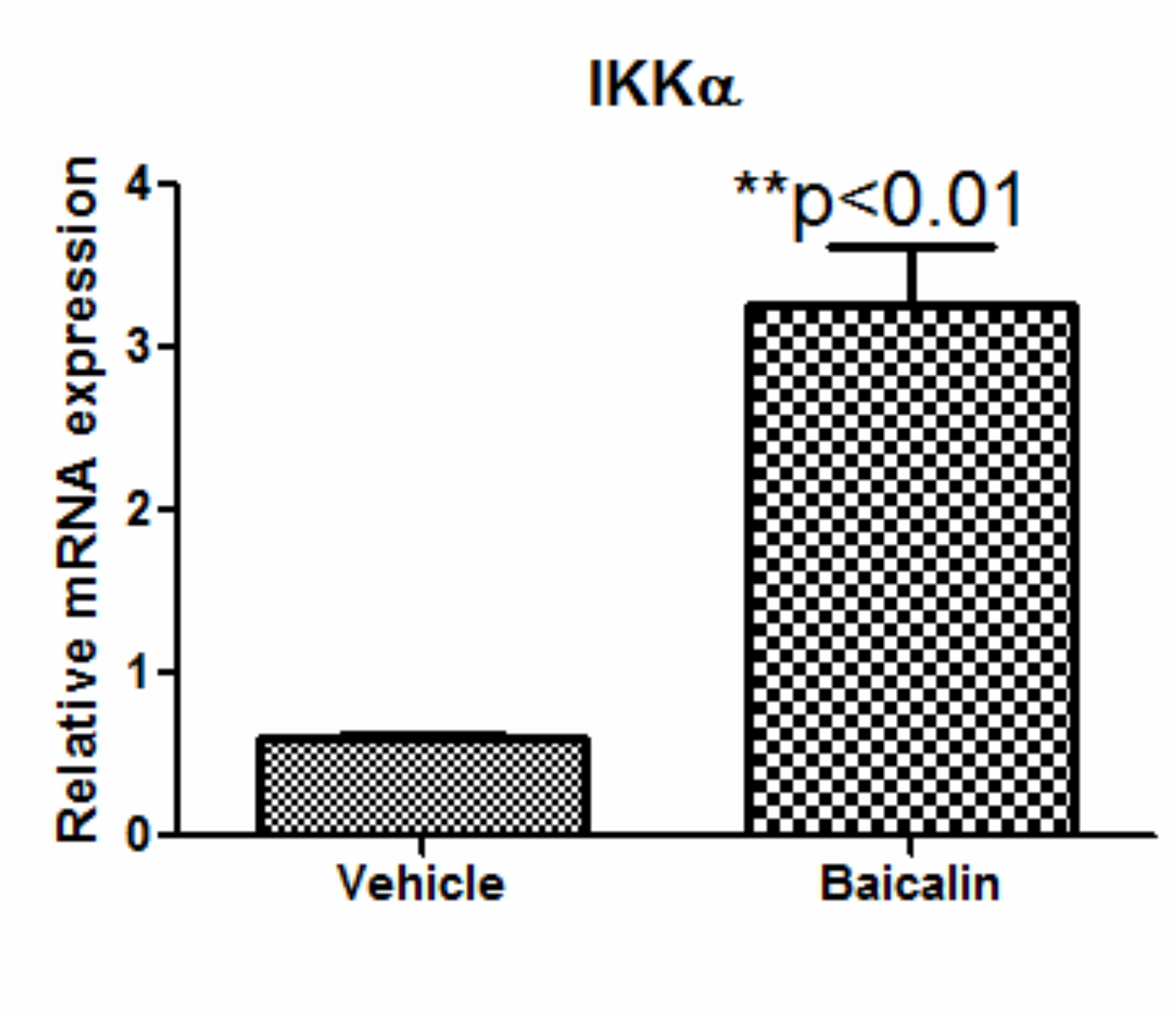


TAM was treated with vehicle or 20μM baicalin for 48 hours and mRNA expression of IKKα was measured by qPCR. Significant increase of IKKα mRNA in baicalin-treated TAM was observed.

**Fig.S4 Baicalin has minimal effect on mRNA level of TRAF2 in TAM**


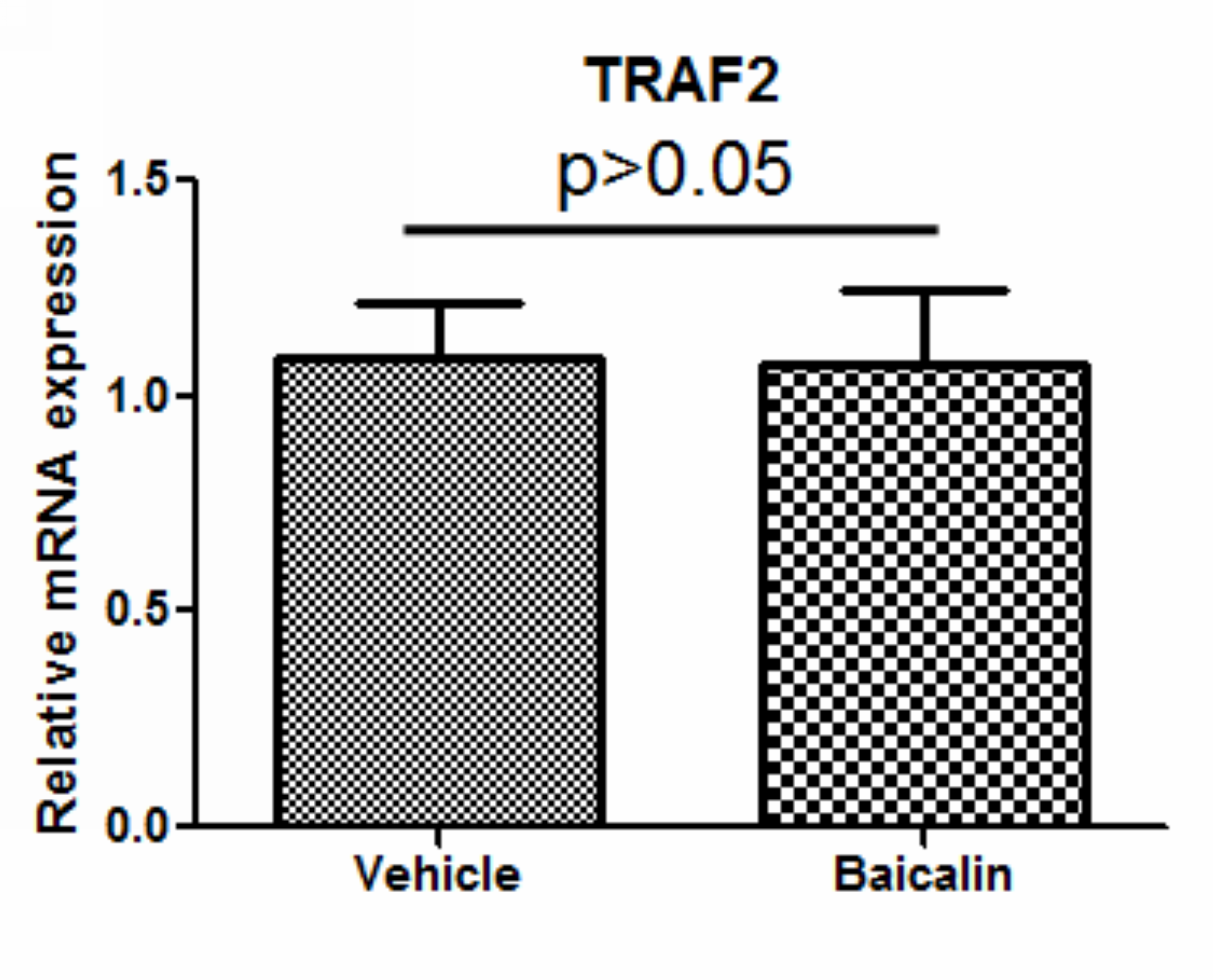


TAM was treated with vehicle or 20μM baicalin for 48 hours and mRNA expression of TRAF2 was measured by qPCR. No significant change of TRAF2 mRNA in baicalin-treated TAM was observed.

**Fig.S5 TRAF2 was ubiquitinated in baicalin-treated TAM**


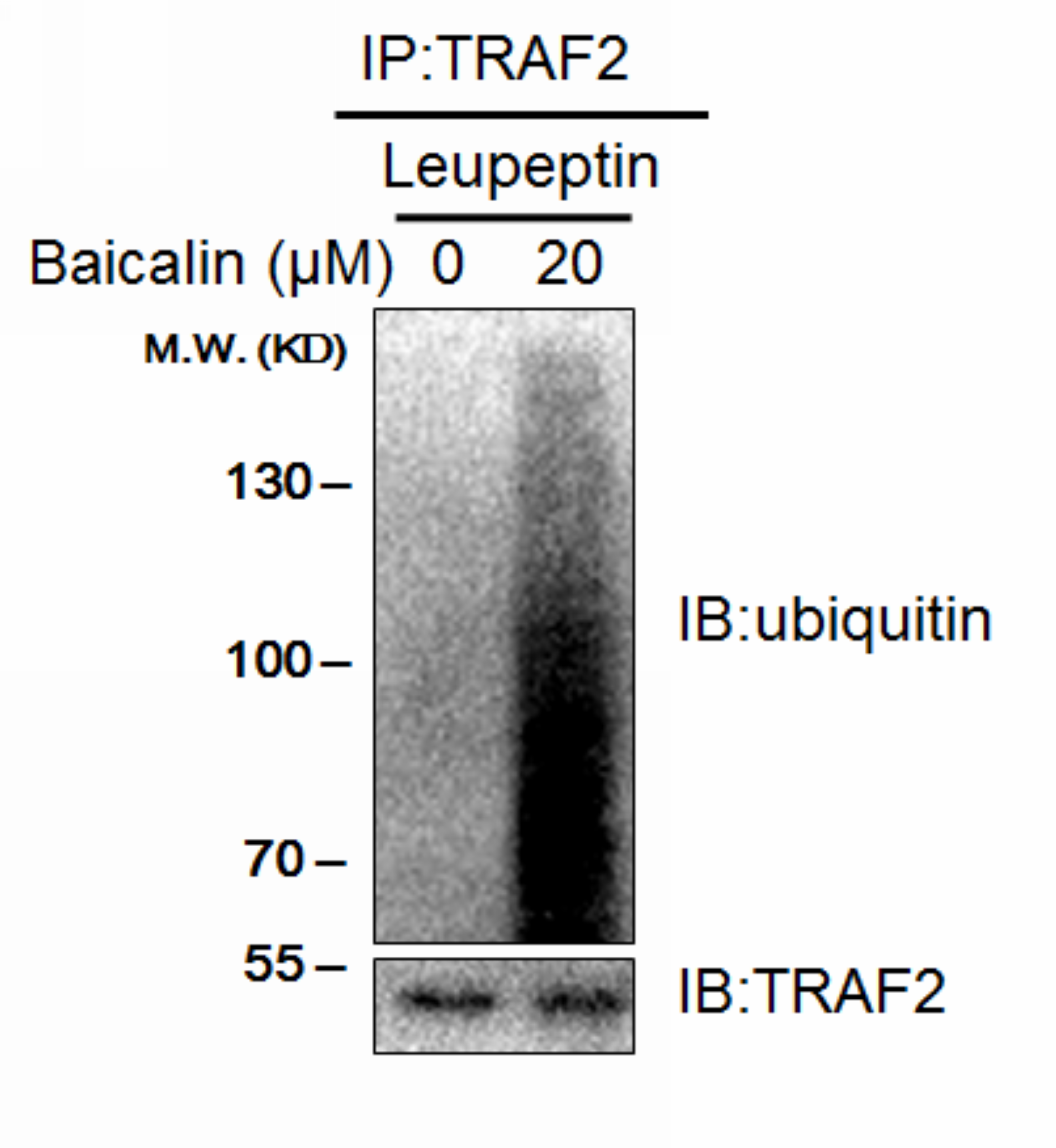


TAM was treated with vehicle or 20μM baicalin for 48 hours in the presence of lysosome inhibitor leupeptin and co-immunoprecipiation assay was conducted with TRAF2 antibody. Immunnoblotting with TRAF2 and ubiquitin antibodies revealed that baicalin could promote ubiquitination of TRAF2 in TAM.

**Fig.S6 TRAF2 was associated with autophagosome**


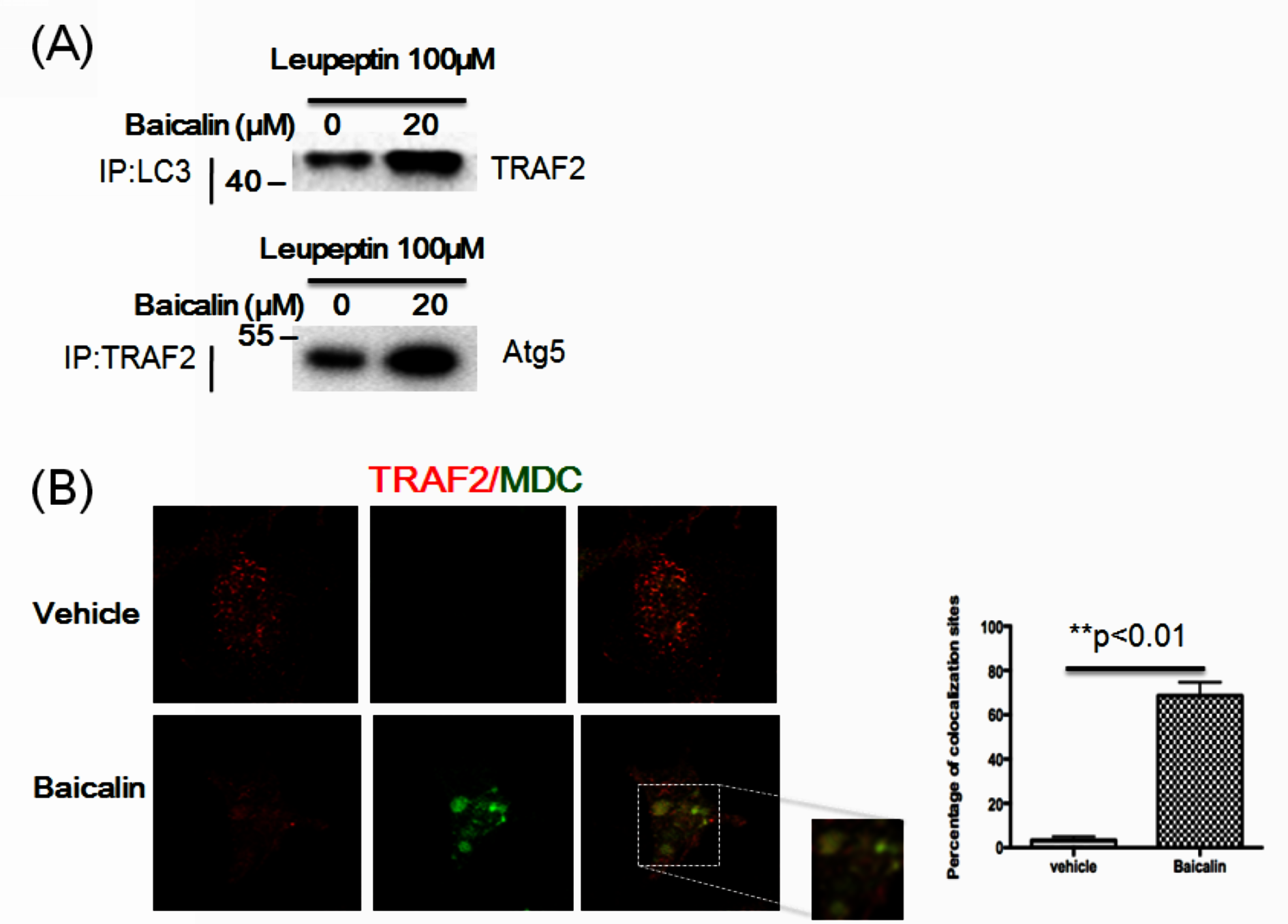


(A) TAM was treated with or without baicalin in the presence of lysosome inhibitor leupeptin. Co-immunoprecipitation assay was conducted with LC3 and TRAF2 antibody and association of TRAF2 with LC3 and Atg5 were detected by immunoblotting. (B) TAM treated with or without baicalin was fixed and stained with TRAF2 antibody and monodansylcadaverine. It was observed that treatment of baicalin could increase the co-localisation of MDC-positive compartment with TRAF2 (Magnification: 60x).

**Fig.S7 RelB and LC3 expressions were increased in *in vivo* liver tissue of HCC bearing mice.**


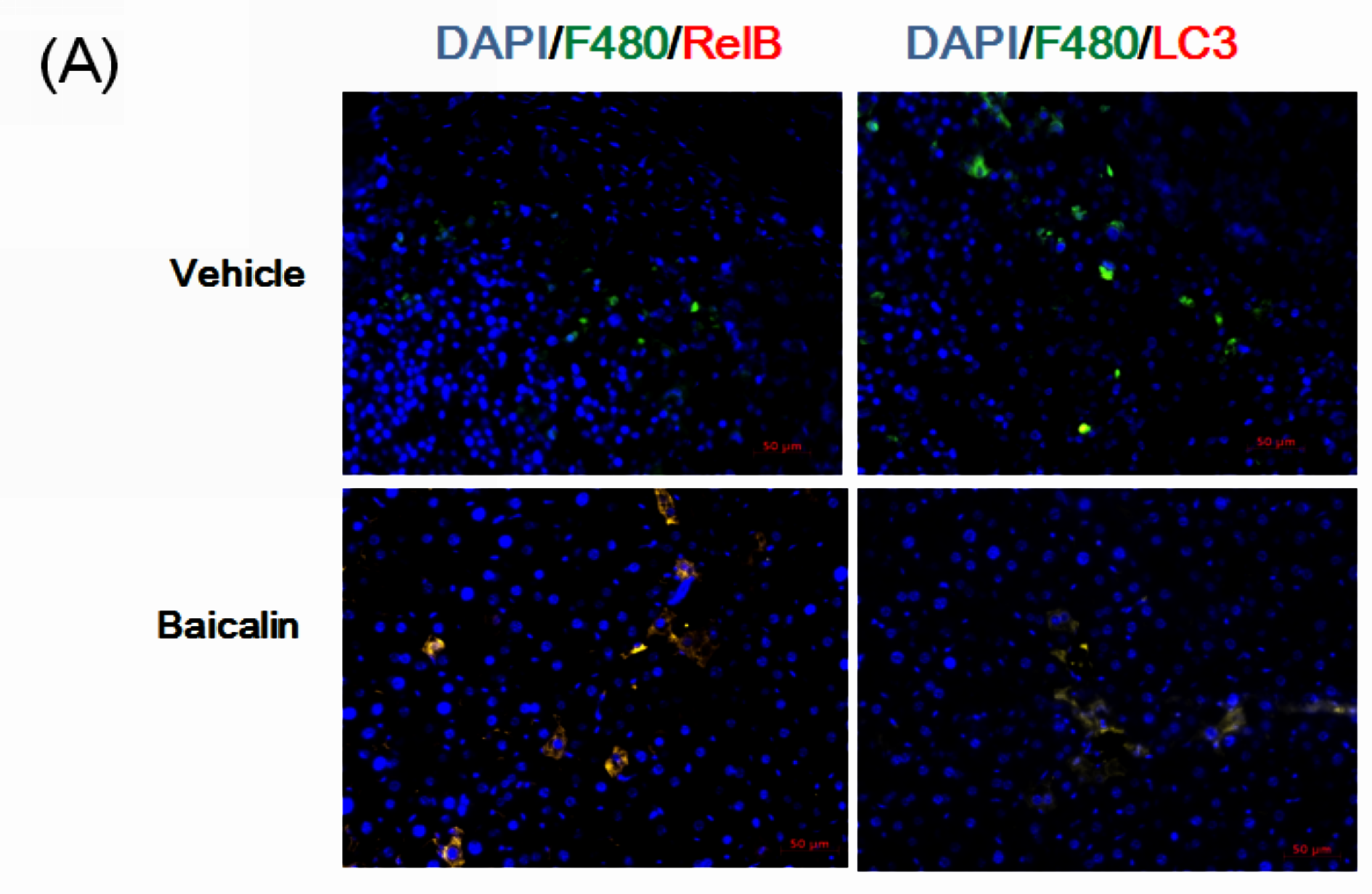


Baicalin treatment induced RelB expression and autophagy in TAMs within liver of HCC-bearing mice. Antibodies against RelB and LC3 were used to co-stain with F480 in frozen sectioned-liver tissue. Increase of RelB and LC3-positive cells all over the liver of baicalin-treated mice was observed (p<0.01) (Scale bar, 50µm).
