# Supplementary material for: Autophagy-induced RelB/p52 activation mediates tumour-associated macrophage repolarisation and suppression of hepatocellular carcinoma by natural compound baicalin
Source: Cell Death Dis. 2015 Oct 22;6(10):e1942–. doi: 10.1038/cddis.2015.271 (PMC4632300; doi:10.1038/cddis.2015.271)
Supplement: Supplementary Methods [file cddis2015271x2.docx]

**Supplemental Methods**

***Isolation of Bone Marrow Derived Monocytes (BMDMs) and macrophage differentiation***

BMDMs culture was prepared using Ficoll density gradient centrifugation procedure. BMDMs collected from femurs and tibias of 6- to 8-week-old C57Bl/6 mice were cultured with complete RPMI-1640 medium (supplemented with 10% Fetal Bovine Serum and 1% Penn/Strep) in the presence of murine M-CSF (10 ng/ml) for 7 days to allow differentiation of monocytes into macrophages (MΦ). MΦs were cultured in complete RPMI-1640 medium in the presence of IFNγ (10 ng/ml) to induce polarisation to M1-like MΦs; while MΦs were exposed to IL4 (20 ng/ml) to programme to M2-like MΦs.

***Generation of Tumour Associated Macrophages (TAM)***

TAMs were generated with HCC tumour cell culture supernatant (TSN). Isolated BMDMs were subject to complete RPMI-1640 medium containing 30% TSN for 7 days to allow differentiation of monocytes to TAM.

***Co-Culture of TAM with HCC cells***

Co-culture of TAM with HCC cells was achieved with Falcon cell culture insert (0.8μm, Corning, New York, USA). HCC cells were cultured in receiving chamber and pre-treated TAM was seeded into culture insert and allowed co-incubation for indicated time slot. Inserts were then removed and HCC cells were subjected to further analysis.

**RNA interference**

All RNAi analysis was performed using FlexiTube siRNA (Qiagen). The macrophage cells were seeded on 6-well plate at a density of 50% confluence in medium without antibiotics, supplemented with 1% FBS overnight. After one night of incubation, the cells were replaced with medium that pre-mixed with 1nmol of siRNA and 10μM transfection reagent (Lipofectamine RNAiMAX; Invitrogen), based on manufacturer’s instructions. The transfection efficiency was assessed by monitoring the expression of targeted genes in transfected cells via immunoblotting.

***Immunofluorescence staining***

For immunofluorescence staining, the cells were fixed in 4% paraformaldehyde and stained with anti-RelB, LC3, p62, Lysotracker and TRAF2. Before counter-stained with DAPI, the cells were further stained with Alexa Fluor 488 or 568-conjugated secondary antibody. Images were acquired by fluorescence confocal microscopy (Carl Zeiss LSM 510 Meta, Germany).

***Flow cytometry analysis***

Cells were trypsinized and washed with PBS. Cells were incubated with anti-mouse CD11b, Ly6C, F4/80, CD86 and CD206 antibody for 15 minutes. Unspecific isotypes were used as controls. The stained cells were washed and analysed by flow cytometry (BD Canto II, New Jersey, USA).

***Quantitative real time PCR***

Total RNA was extracted with TRIzol reagent (Invitrogen, California, USA). Reverse-transcription reaction was conducted with PrimeScript RT Reagent kit (Takara, Japan). Quantitative real time PCR was performed with SYBR Green PCR Kit (Qiagen, Germany) and 1μM primers using Light Cycler 480 real-time PCR system (Roche, Basel, Switzerland). The mouse primers pair sequences are attached in supplementary material and β-actin serves as internal control (Table S1). Relative expression of target genes against β-actin was expressed as 2^-ΔCt^ and fold differences was calculated as expressed mRNA of baicalin-treated samples against untreated samples.

***Immunoblotting***

Total protein was extracted via radio-immuno-precipitation lysis (RIPA) buffer supplemented with complex cocktail proteinase inhibitor (Roche, Basel, Switzerland) and phosphatase inhibitor (1mM Na_3_VO_4_ and 1mM NaF) and concentration was determined using BSA as standard. The equal amounts of proteins were separated on SDS-PAGE, and transferred onto polyvinylidenedifluoride membranes (PVDF, Millipore).The membrane was blocked in 5% of BSA in buffer containing Tris (10mmol/L, pH 7.4), NaCl (150mmol/L) and Tween 20 (1%), followed by primary antibody incubation at 4^0^C overnight. The membrane was washed in Tris-NaCl buffer an incubated with HRP–labelled secondary antibody for 2 hours. Signals were detected via ECL advanced kit (GE Healthcare, UK) and visualized using chemiluminescence imaging system (Bio-Rad, California, USA). The images were acquired and analysed with ImageJ.

***Co-immunoprecipitation assay***

Co-immunoprecipitation assay was performed using Pureproteome protein G magnetic beads (Millipore, [Massachusetts](https://en.wikipedia.org/wiki/Billerica,_Massachusetts), USA), according to the manufacturer’s instructions. Briefly, the cell proteins were extracted with NP-40 lysis buffer (Invitrogen, California, USA) supplemented with complex cocktail proteinase inhibitor (Roche, Basel, Switzerland). The target protein was precipitated through binding with target antibody pre-bound magnetic bead and removed by eluting with PBS supplemented with 0.1% tween 20. Isolated protein was further denatured by heating in 95^0^C with loading buffer. The targeted protein and other interacted proteins were separated in SDS-PAGE and protein expressions were visualized using chemiluminescence imaging system (Bio-Rad, California, USA).The input lysates serves as loading control of the targeted protein.

***Cytokine analysis by cytometric bead array.***

Culture supernatants of untreated and baicalin-treated macrophages were collected after 48 hours. Determination of cytokine expression (IL6, TNF-α and IL10) was performed using cytometric bead array flex set kit by flow cytometry (BD Canto II, New Jersey, USA) according to the manufacturer’s instructions (BD biosciences, UK).
