# Supplementary material for: Autophagy-induced RelB/p52 activation mediates tumour-associated macrophage repolarisation and suppression of hepatocellular carcinoma by natural compound baicalin
Source: Cell Death Dis. 2015 Oct 22;6(10):e1942–. doi: 10.1038/cddis.2015.271 (PMC4632300; doi:10.1038/cddis.2015.271)
Supplement: Supplementary Table 1 [file cddis2015271x3.docx]

**Supplemental Table S1 Sequence of mouse primer pairs used in this study**

| Gene Name | Forward | Reverse |
| --- | --- | --- |
| TNF-α | 5’-CTGTAGCCCACGTCGTAGC-3’ | 5’-TTGAGATCCATGCCGTTG-3’ |
| IL12 | 5’-TACTAGAGAGACTTCTTCCACAACAAGAG-3’ | 5’-TCTGGTACATCTTCAAGTCCTCATAGA-3’ |
| IL10 | 5’-ATTTGAATTCCCTGGGTGAGAAG-3’ | 5’-CACAGGGGAGAAATCGATGACA-3’ |
| Arginase1 | 5’-CTCCAAGCCAAAGTCCTTAGAG-3’ | 5’-AGGAGCTGTCATTAGGGACATC-3’ |
| CCL9 | 5’-CCTCAGCCTGCTGGTTCTCT-3’ | 5’-CAGCAGTCTTCAGCATCATTGG-3’ |
| CXCL12 | 5’-CAGAGCCAACGTCAAGCA-3’ | 5’-AGGTACTCTTGGATCCAC-3’ |
| IKKα | 5’-CCATTCACTATTCTGAGGTTGGTGTC-3’ | 5’-TACTGGAGGGGTTACTGTGCCTTC-3’ |
| RelB | 5’-CCGAGCTAGGGGCCTTGGGTTCC -3’ | 5’-AGCTCGATGGCGGGCAGGGTCTTG-3’ |
| TRAF2 | 5’-TTCGGCCTTTCCAGATAACGC -3’ | 5’-CCTTCCAAGTGCATCCATCATTG -3’ |
| β-actin | 5’-CCTGAGGCTCTTTTCCAGCC-3’ | 5’-TAGAGGTCTTTACGGATGTCAACGT-3’ |
